# Glioblastoma in adults with prior extracranial cancer: a Czech retrospective multicentre study

**DOI:** 10.3389/fonc.2026.1897811

**Published:** 2026-07-29

**Authors:** Ondrej Kalita, Tomas Kazda, Silvia Tomoszkova, Radim Jancalek, Marek Slachta, Dominik Fric, Tomas Krejci, Pavla Kourilova, Jakub Cvek, Martin Dolezel, Lumir Hrabalek, Radim Lipina

**Affiliations:** 1 Department of Neurosurgery, Faculty of Medicine and Dentistry, Palacký University Olomouc, University Hospital Olomouc, Olomouc, Czechia; 2 Department of Health Care Science, Faculty of Humanities, T. Bata University in Zlin, Zlín, Czechia; 3 Department of Radiation Oncology, Faculty of Medicine, Masaryk University and Masaryk Memorial Cancer Institute, Brno, Czechia; 4 Department of Neurosurgery, Faculty of Medicine, University of Ostrava, University Hospital Ostrava, Ostrava, Czechia; 5 Department of Neurosurgery, Faculty of Medicine, Masaryk University and St. Anne’s University Hospital in Brno, Brno, Czechia; 6 Institute of Molecular and Translational Medicine, Faculty of Medicine and Dentistry, Palacky University Olomouc, Olomouc, Czechia; 7 Department of Oncology, Faculty of Medicine, University of Ostrava, University Hospital Ostrava, Ostrava, Czechia; 8 Department of Oncology, Faculty of Medicine and Dentistry, Palacký University Olomouc, University Hospital, Olomouc, Czechia

**Keywords:** cancer survivorship, extracranial cancer, glioblastoma, multicentre study, retrospective cohort, second primary malignancy

## Abstract

**Background:**

Patients with a history of extracranial cancers (EXCA) who develop new intracranial lesions are commonly presumed to harbour brain metastases, yet primary glioblastoma (GBM) can also arise *de novo* in cancer survivors. The clinical course, molecular features, and treatment outcomes of GBM in this population remain incompletely characterised.

**Methods:**

We retrospectively analysed adult patients with concurrent prior solid EXCA and supratentorial GBM treated at three Czech neuro-oncology centres between January 2010 and December 2022. Patients with hematologic malignancies or needle-biopsy-only diagnoses were excluded. Demographic, clinical, molecular (IDH, MGMT), surgical (RANO RESECT 2022 extent-of-resection classification), and treatment data were collected and related to progression-free survival (PFS) and overall survival (OS) using Kaplan–Meier estimation, log-rank tests, and Cox regression.

**Results:**

Of 1,182 patients operated for GBM, 48 (4.0%) had a documented prior solid EXCA. Median age at EXCA diagnosis was 57.5 years (range 21–82); median age at GBM diagnosis was 65 years (range 46–82). The median interval between diagnoses was 5.7 years (range 0.1–27.3). All 48 GBMs were IDH-wildtype; MGMT promoter status was assessable in 23 cases (17 unmethylated, 6 methylated), of which 22 had follow-up data available for survival analysis. Median PFS was 8.4 months (95% CI 7.6–27.3) and median OS was 10.6 months (95% CI 5.7–16.8). MGMT methylation (mOS 29.3 vs 12.0 months; p = 0.037), higher Karnofsky score, more radical surgical resection, and completion of the Stupp protocol (mOS 19.2 vs 2.5 months for no oncotherapy; p = 0.019) were each significantly associated with longer OS.

**Conclusions:**

A new brain lesion in a cancer survivor must not be assumed to be metastatic without histological confirmation. When patients with GBM after prior EXCA are eligible for standard radical surgery and Stupp-protocol oncotherapy, survival outcomes are comparable to those reported for sporadic GBM. Standard treatment protocols are appropriate for this population; a history of extracranial cancer is not in itself an adverse prognostic factor.

## 1 Introduction

Patients with a history of extracranial carcinomas (EXCA) who develop intracranial lesions are often presumed to have metastases. In fact, brain metastases comprise ~50–60% of adult intracranial tumours, most commonly from lung, breast, melanoma and renal cell carcinoma (1). Brain metastases have been found in 10–50% of patients with cancer history with the cerebral hemispheres being involved in 80% of cases (2). Current progress in local treatments (surgery, radiotherapy) and mainly systemic treatments (chemotherapy, targeted treatment, immunotherapy, etc.) augments extracranial disease control and increases the life expectation for these patients who are candidates for subsequent second primary malignancy including glioblastoma (GBM). When neurologic deficits or epileptic seizures appear, brain metastases evolution should be considered, and an urgent imaging is required. Silent, asymptomatic brain metastases are detected only rarely. The benefits of the surgery, hippocampus-avoiding whole brain radiotherapy/stereotactic radiotherapy, or systemic treatment is considered (3, 4).

However, case reports and series from the past two decades document that primary high-grade gliomas (HGG), especially GBM, can also develop *de novo* in these surviving patients (5–8).

The aim of this retrospective multicentric study is to evaluate the incidence of concurrent tumours in GBM patients, to assess the relationship between previous EXCA staging and GBM evaluation as well as to provide real-world description of treatment efficiency in this specific cohort of patients.

## 2 Materials and methods

### 2.1 Patients

This retrospective multicentre cohort study was conducted at three cooperating neuro-oncology centres in the Czech Republic: University Hospital Olomouc (Department of Neurosurgery and Spinal Surgery); Masaryk Memorial Cancer Institute Brno (Department of Radiation Oncology); and University Hospital Ostrava (Department of Neurosurgery). Data of all glioma patients treated at these centres are collected regularly and prospectively as part of the routine maintenance of the national database of operated patients. This work focuses on adult patients with concurrent history of both solid EXCA and supratentorial GBM who underwent resection and oncotherapy between January 2010 and December 2022. We excluded patients with a history of hematologic malignancy and with intracranial tumours diagnosed only by a needle biopsy. This database was also used to collect information about the GBM cytogenetic features, treatment strategy, imaging, clinical conditions and disease course. Finally, the details gathered were related to the patient´s gender, the type and treatment of the previous EXCA, the age at the time of EXCA and GBM diagnosis, the EXCA status in time of GBM diagnosis and during GBM treatment, GBM IDH status, GBM MGMT status, Karnofsky score before GBM surgery, radicality of GBM surgery and the GBM therapy type, life span from EXCA and GBM diagnosis and final cause of patient death if any.

Almost all of these variables were categorical, except of the patient´s age at EXCA, the age at GBM and the interval between EXCA and GBM, which were treated as a continuous variable.

The local relevant multidisciplinary tumour board determined a treatment strategy for all patients with EXCA history considering the potential of brain metastasis.

Regarding all brain tumours, clinicians at all three centres strove to perform as safe and radical resection as possible. An adjuvant standard aggressive oncotherapy (Stupp protocol) was supposed to be applied to all GBM patients (9). All patients underwent early postsurgical MRI (within 72 h) to determine resection radicality. The degree of removal of the original tumour volume was sorted according to RANO resect group new classification for extent of resection in GBM 2022: Class 1: supramaximal Contrast Enhanced (CE) resection (no CE residual tumour + ≤5 cm^3^ nonCE residual tumour), Class 2A: complete CE resection ((no CE residual tumour + >5 cm^3^ nonCE residual tumour), Class 2B: near total CE resection (≤1 cm^3^ CE residual tumour), Class 3A: subtotal CE resection (≤5 cm^3^ CE residual tumour), Class 3B: partial CE resection (>5 cm^3^ CE residual tumour), Class 4: biopsy (no reduction of tumour volume) (10).

All of the tumours were histologically classified by local neuropathologists. The former tumour classifications initially used in the clinical setting were converted into WHO 2021 GBM classifications in accordance with current recommendations (11). Following diagnosis, patients underwent periodic checkups with MRI every 3 months until death. Tumour tissue samples were collected in both formalin-fixed, paraffin-embedded (FFPE) forms and fresh-frozen forms.

### 2.2 Investigation of IDH mutation and MGMT promotor methylation

*Isocitrate dehydrogenase* (IDH) mutation and *O6-methylguanine-DNA methyltransferase* (MGMT) promoter methylation status were assessed using standard techniques employed at the three neuro-oncology centres (12–14). The first step was immunohistochemical (ICH) examination of the canonical IDH mutation (anti-IDH1R132H), supplemented with IDH genotyping using Next-Generation Sequencing (Nextera XT kit, Illumina, San Diego, CA, USA), and examination of MGMT promoter methylation using Real-time methylation-specific PCR.

IDH status testing in our centres has been performed regularly since January 2015. GBM patients with prior EXCA without known IDH status were included if at least a minimal amount of tumour tissue was available to complete IDH mutation testing. Even according to the results of our research, which confirmed the WHO recommendations, NGS was performed in patients with gliomas under 55 years of age with ICH negativity for the IDH1 R132H mutation (12).

### 2.3 Statistical methods

Statistical analysis was performed using R, version 4.4.2 (Core Team, 2024). All tests were two-tailed and the significance level was set at p < 0.05. The relationships between independent variables were examined by using Fisher’s exact test, ANOVA test, Kruskal-Wallis test, t-test or Wilcoxon exact test, respectively. The univariate effects of the independent factors on the progress-free survival (PFS = time from GBM diagnosis to radiologic progression/last clinical control) and overall survival (OS = time from GBM diagnosis to death) were studied using the log-rank test and/or Cox proportional hazard regression and visualised by Kaplan-Meier curves.

### 2.4 Handling of missing data

**Figure 1:**
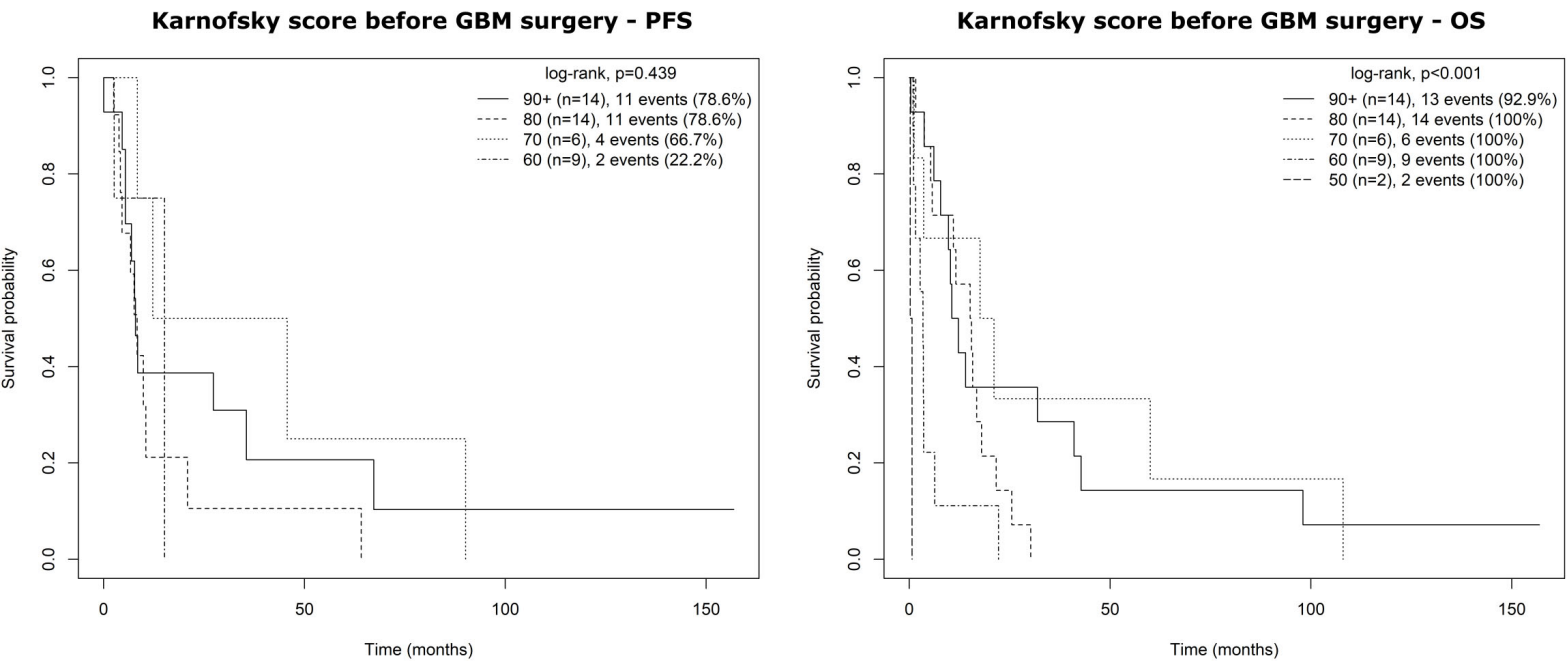
Kaplan–Meier estimates by preoperative Karnofsky Performance Score. PFS (left) and OS (right) stratified by preoperative Karnofsky Performance Score (KPS). Log-rank p = 0.439 for PFS; p < 0.001 for OS. PFS analysis: n = 44 (the KPS 50 subgroup contained one patient with no events and is not visible on the PFS curve). OS analysis: n = 45.

**Figure 2:**
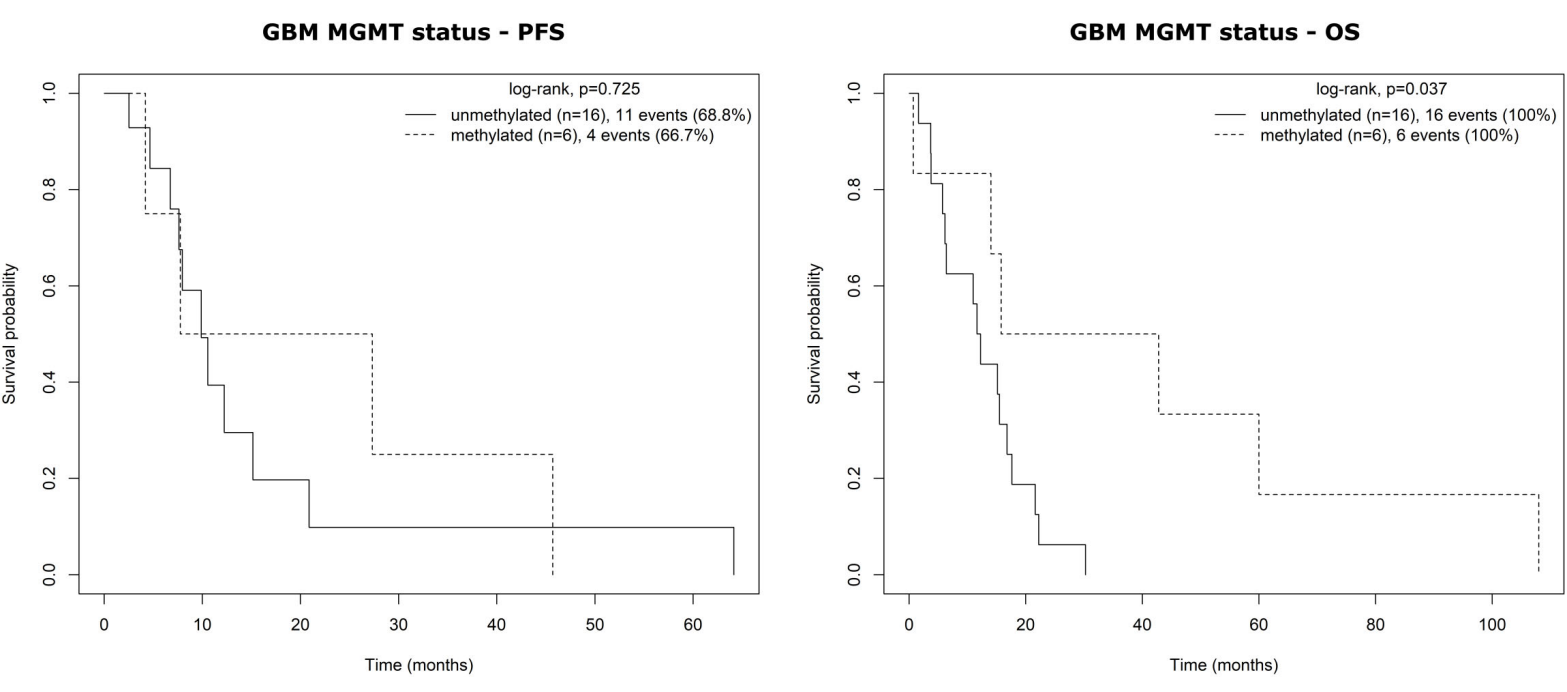
Kaplan–Meier estimates by MGMT promoter methylation status. PFS (left) and OS (right) stratified by MGMT promoter methylation status. Log-rank p = 0.725 for PFS; p = 0.037 for OS. MGMT status was assessable in 23 of 48 tumours; survival analysis was performed on 22 patients with available follow-up (16 unmethylated, 6 methylated).

**Figure 3:**
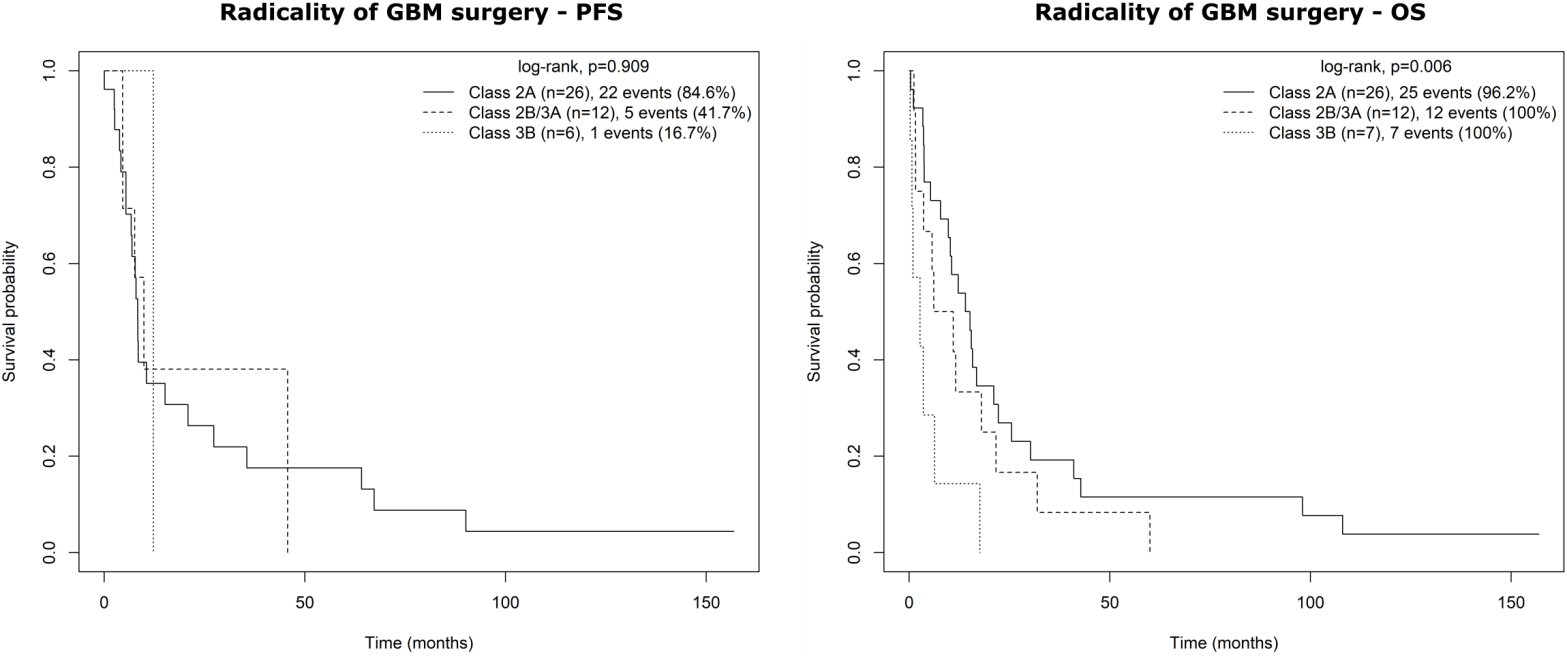
Kaplan–Meier estimates by extent of resection (RANO RESECT 2022). PFS (left) and OS (right) stratified by postoperative residual tumour volume according to the RANO RESECT 2022 classification. Class 2B (near-total contrast-enhancing resection) and Class 3A (subtotal resection) were merged owing to small subgroup size. Log-rank p = 0.909 for PFS; p = 0.006 for OS. PFS analysis: n = 44; OS analysis: n = 45.

**Figure 4:**
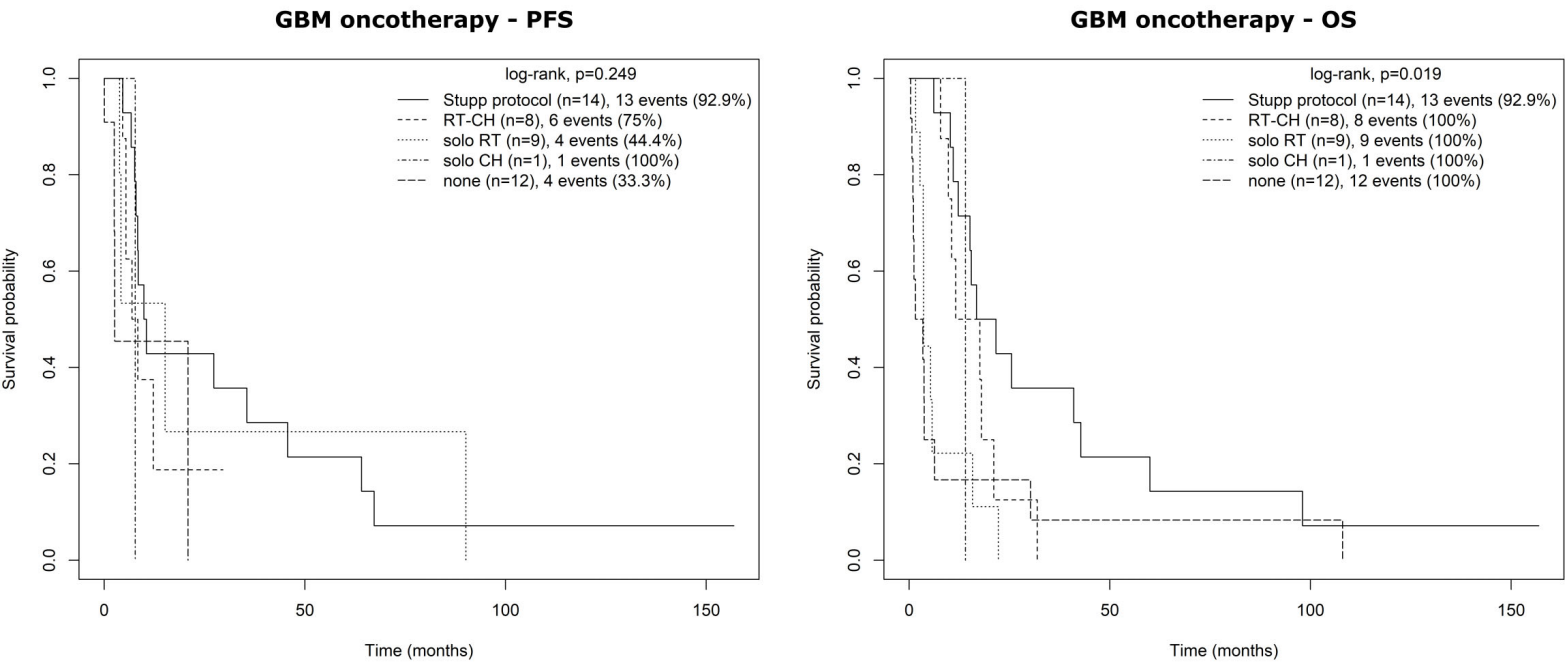
Kaplan–Meier estimates by GBM adjuvant oncotherapy. Progression-free survival (PFS, left) and overall survival (OS, right) stratified by adjuvant oncotherapy regimen. Stupp protocol denotes concomitant chemoradiotherapy followed by at least three cycles of adjuvant temozolomide. Log-rank p = 0.249 for PFS; p = 0.019 for OS. PFS analysis: n = 44. OS analysis: n = 44 (one patient with OS data lacked a documented therapy classification and was excluded from this comparison). CH, chemotherapy; RT, radiotherapy.

Survival analyses were performed on a complete-case basis. Progression-free survival could be assessed in 44 of 48 patients (3 patients had no available follow-up, and 1 additional patient had overall survival data but no evaluable PFS event). Overall survival could be assessed in 45 of 48 patients (3 patients with no available follow-up). MGMT promoter methylation status was assessable in 23 of 48 tumour samples; the remaining 25 samples had insufficient tissue quality for reliable methylation-specific PCR. Subgroup denominators in **Tables 1**–**3** and in **Figures 1**–**4** reflect the number of patients with both the variable and the corresponding survival endpoint available.

**Table 1:** Characteristics of extracranial cancers.

Previous primary Cancers	Male reproductive system cancer	11/47 (23.4%)
	GIT carcinoma	10/47 (21.3%)
	Urinary tract carcinoma	8/47 (17%)
	Skin carcinoma	6/47 (12.8%)
	Breast carcinoma	5/47 (10.6%)
	Lung carcinoma	4/47 (8.5%)
	Gynaecological carcinoma	2/47 (4.3%)
	Oral cavity carcinoma	1/47 (2.1%)
	*Missing primary cancer*	*1*
EXCA therapy	Surgery	17/48 (35.4%)
	surgery + CH	9/48 (18.8%)
	surgery + RT	10/48 (20.8%)
	surgery + immunotherapy	1/48 (2.1%)
	surgery + biologic therapy	1/48 (2.1%)
	RT+CH	3/48 (6.2%)
	CH	2/48 (4.2%)
	RT	4/48 (8.3%)
	no therapy/following	1/48 (2.1%)
EXCA status in time of GBM diagnosis	in remission	38/48 (79.2%)
	Stable	8/48 (16.7%)
	in progression	2/48 (4.2%)
EXCA status during GBM therapy	in remission	36/44 (81.8%)
	Stable	2/44 (4.5%)
	in progression	6/44 (13.6%)
	*Missing*	*4*

EXCA, extracranial carcinoma; CH, chemotherapy; RT-CH, radiotherapy + adjuvant chemotherapy; RT, radiotherapy.

**Table 2:** Histological and clinical characteristics of patient dataset.

Characteristics	Variable	Value
GBM MGMT status	unmethylated	17/23 (73.9%)
	methylated	6/23 (26.1%)
	*missing*	*25*
Karnofsky score before GBM surgery	90+	15/48 (31.2%)
	80	14/48 (29.2%)
	70	8/48 (16.7%)
	60	9/48 (18.8%)
	50	2/48 (4.2%)
Radicality of GBM surgery	Class 2A	28/48 (58.3%)
	Class 2B	2/48 (4.2%)
	Class 3A	11/48 (22.9%)
	Class 3B	7/48 (14.6%)

EXCA, extracranial carcinoma; IDH, isocitrate dehydrogenase; MGMT, O6-methylguanine-DNA methyltransferase; CE, Contrast enhancement, Class 2A: complete CE resection (no CE residual tumour + >5 cm^3^ nonCE residual tumour), Class 2B: near total CE resection (≤1 cm^3^ CE residual tumour), Class 3A: subtotal CE resection (≤5 cm^3^ CE residual tumour), Class 3B: partial CE resection (>5 cm^3^ CE residual tumour); Stupp protocol, concomitant chemoradiotherapy + minimally 3 cycles of adjuvant chemotherapy; RT-CH, radiotherapy + adjuvant chemotherapy; RT, radiotherapy; CH, chemotherapy.

**Table 3:** Univariate associations of clinical, molecular, and treatment variables with progression-free survival (PFS) and overall survival (OS) in 48 GBM patients with prior extracranial cancer.

		PFS				OS			
		N	Events	Median (95% CI)	logrank test pvalue	N	Events	Median (95% CI)	Logrank test pvalue
All patients		44	28 (63.6%)	8.4 (7.6,27.3)		45	44 (97.8%)	10.6 (5.7,16.8)	
sex	M	30	19 (63.3%)	8.5 (8,45.7)	0.758	31	31 (100%)	10.6 (3.8,21.1)	0.740
	F	14	9 (64.3%)	7.8 (4.6,NA)		14	13 (92.9%)	11.9 (5.7,42.8)	
EXCA status in time of GBM diagnosis	in remission	37	25 (67.6%)	8.4 (7.6,27.3)	0.927	37	36 (97.3%)	11 (6.4,17.6)	0.442
	stable	5	2 (40%)	NA (4.7,NA)		6	6 (100%)	4.9 (0.4,NA)	
	in progression	2	1 (50%)	20.9 (NA,NA)		2	2 (100%)	15.9 (1.6,NA)	
EXCA status during GBM therapy	in remission	36	21 (58.3%)	9.9 (7.8,35.6)	0.438	36	35 (97.2%)	12 (5.7,17.6)	0.377
	stable	2	1 (50%)	4.7 (NA,NA)		2	2 (100%)	4.9 (3.7,NA)	
	in progression	6	6 (100%)	8 (5.5,NA)		6	6 (100%)	9.4 (3.6,NA)	
GBM MGMT status	unmethylated	16	11 (68.8%)	9.9 (7.63,NA)	0.725	16	16 (100%)	12 (6.2,21.6)	0.037
	methylated	6	4 (66.7%)	17.5 (4.21,NA)		6	6 (100%)	29.3 (14.0,NA)	
Karnofsky score before GBM surgery	90+	14	11 (78.6%)	8 (5.5,NA)	0.439	14	13 (92.9%)	11.4 (9.8,98.0)	<0.001
	80	14	11 (78.6%)	8.4 (4.6,NA)		14	14 (100%)	15.3 (11.0,35.0)	
	70	6	4 (66.7%)	29 (8.4,NA)		6	6 (100%)	19.4 (3.6,NA)	
	60	9	2 (22.2%)	15.2 (2.7,NA)		9	9 (100%)	3.4 (1.6,NA)	
	50	1	0 (0%)	NA (NA,NA)		2	2 (100%)	0.5 (0.3,NA)	
Radicality of GBM surgery	Class 2A	26	22 (84.6%)	8.4 (6.7,27.3)	0.909	26	25 (96.2%)	14.6 (9.8,25.5)	0.006
	Class 2B/3A	12	5 (41.7%)	9.9 (4.7,NA)		12	12 (100%)	8.6 (3.6,NA)	
	Class 3B	6	1 (16.7%)	12.2 (NA,NA)		7	7 (100%)	2.7 (0.7,NA)	
GBM oncotherapy	Stupp protocol	14	13 (92.9%)	10.2 (8.4,67.3)	0.249	14	13 (92.9%)	19.2 (15.2,98.0)	0.019
	RT-CH	8	6 (75%)	7.7 (5.5,NA)		8	8 (100%)	14.6 (10.6,NA)	
	solo RT	9	4 (44.4%)	15.2 (4.2,NA)		9	9 (100%)	3.6 (3.5,NA)	
	solo CH	1	1 (100%)	7.8 (NA,NA)		1	1 (100%)	14 (NA,NA)	
	none	12	4 (33.3%)	2.7 (2.5,NA)		12	12 (100%)	2.5 (1.09,NA)	

Median values with 95% confidence intervals; p-values from log-rank tests. Bold p-values indicate statistically significant associations (p < 0.05). Denominators (N) reflect the number of patients with both the variable and the corresponding survival endpoint available. Missing data: 4 patients for PFS (3 with no available follow-up, 1 with OS data but no evaluable PFS event); 3 patients for OS. MGMT status: 25 tumours not assessable. EXCA status during GBM therapy: 4 not documented. GBM oncotherapy: 1 patient with OS data lacked a documented therapy classification.

## 3 Results

In total, there were 1182 patients who underwent surgery for GBM and 48 (4.0%) out of them had a history of solid EXCA, with characteristics summarised in **Table 1**.

The age of patients at the time of EXCA varies from 21 to 82 years with a median of 57.5 years (IQR 50.8-69). The age of patients at the time of GBM diagnosis ranged between 46 and 82 years, with a median age of 65 years (IQR 58.75-72). The median of the interval between the EXCA and GBM diagnosis was 5.7 years (IQR 2.6-11.1) with the range from 0.1 to 27.3 years. With increasing age at EXCA diagnosis, the time to subsequent GBM diagnosis decreases (Spearman correlation coefficient = -0.53, p < 0.001).

Histological, clinical and treatment characteristics of GBM and relations between GBM and EXCA are presented in **Table 2**.

Canonical IDH mutation was not found in all 48 GBM patients, even retrospectively. In 25 cases, MGMT promoter methylation could not be reliably assessed due to insufficient quality of the available samples.

As expected from sex-specific tumour biology, the spectrum of previous cancers differed between men (predominantly prostate and urinary tract carcinomas) and women (predominantly breast and gynaecological carcinomas); this difference reflects sex-specific tumour distribution rather than a biologically meaningful association. The interval between EXCA and GBM diagnosis was descriptively longer in women than in men (median 12.0 vs 5.05 years).

The expected relationships between the clinical condition, patient age and the outcomes were confirmed (**Table 3**). The patient’s overall condition (assessed by the Karnofsky score (KS)) and the younger age are significantly connected to the radicality of the GBM surgery (Fisher’s exact test, p= 0.01) and with the subsequent aggressive GBM therapy (Fisher’s exact test, p =0.015). Patients with higher Karnofsky score underwent more radical surgery as well as more aggressive and effective oncologic treatment (Stupp protocol, RT-CHT). Preoperative Karnofsky score was significantly associated with OS (log-rank p < 0.001) but not with PFS (p = 0.439) (**Figure 1**). A sensitivity analysis using the standard clinical dichotomisation of KPS at ≥ 80 versus < 80 is provided in **Supplementary Figure S4**.

Of the 48 patients, 41 patients succumbed to GBM, 3 for other reasons, one is still alive and the survival data are missing for three patients (**Table 3**). The PFS median (mPFS) was 8.4 months (95% CI 7.6-27.3) and median OS (mOS) was 10.6 months (95% CI 5.7-16.8). A longer OS was found in the patients with MGMT methylation (mOS = 29.3 months, n = 6) in comparison to MGMT unmethylated (mOS = 12.0 months, n = 16; log-rank test, p = 0.037; HR for unmethylated = 3.58, p = 0.049) (**Figure 2**).

According to the postoperative residual GBM volume, mOS was 14.6 months in patients with Class 2A (9.8, 25.5), 8.6 months in patients with Class 2B/3A(3.6, NA), 2.7 months in patients with Class 3B (0.7, NA) (**Figure 3**). In patients with aggressive adjuvant oncotherapy by Stupp protocol, mOS was 19.2 months (15.2,98.0). Other oncologic strategies resulted in reduced mOS (**Table 3**; **Figure 4**).

Neither sex (log-rank p = 0.758 for PFS, p = 0.740 for OS) nor the disease status of the prior extracranial cancer — either at the time of GBM diagnosis (p = 0.927 for PFS, p = 0.442 for OS) or during GBM oncotherapy (p = 0.438 for PFS, p = 0.377 for OS) — was significantly associated with survival (**Supplementary Figures S1**-**S3**).

## 4 Discussion

GBM following EXCA is uncommon. Based on analyses from our three neuro-oncologic centres, out of the 1182 patients who underwent resection for GBM, only 4% have experienced previous solid EXCA. The age of our patients at the time of EXCA diagnosis varied from 21 to 82 years with median age 57.5 years, which corresponds to the age distribution of sporadic GBM. The patient age at the time of GBM ranged between 46 and 82 years with median age 65 years. The mean interval between EXCA and GBM diagnosis was 5.7 years, ranging from 0.1 to 27.3 years. The interval was longer for women (12 years contrary to 5.05 years in men) and with increasing age at EXCA diagnosis, the time to GBM diagnosis decreases.

Large registry data show that only a very small fraction of cancer survivors develops a subsequent primary brain tumour. A prior SEER-based analysis found that among 6 million patients with a non-CNS malignancy, only 5,212 (≈0.09%) had later a primary malignant brain tumour (7). The most frequent antecedent cancers in that cohort were prostate (28%), breast (15%), and cutaneous melanoma (11%) (7). Certain prior cancers were associated with notably higher relative risks (e.g. ALL, renal cell, thyroid) and lower risks after others (e.g. breast, larynx) (7). Survivors of acute lymphoblastic leukaemia had a 4.7-fold increased standardised incidence ratio (SIR) for developing a brain tumour. Overall, most of these secondary primary brain tumours are gliomas (94%), with 68.4% being GBM and only small percentages of other subtypes (anaplastic astrocytoma, oligodendroglioma, etc.) (7). The latest SEER-based analysis of patient data for brain cancers as their first or second primary malignancy between 2000 and 2019 revealed that of 42,726 patients, only 1,189 (2.78%) had EXCA (8). The most frequent EXCA were genitourinary (40.4%), followed by breast (13.6%), hematologic (11.4%), and GIT malignancies (11.3%). Again, GBM dominated among glioma histologic subtypes. Contrary, in an institutional series of primary glioma patients, the prevalence of prior cancers was higher. Zacharia et al. reported 170 of 2,164 GBM patients (7.9%) with a history of another malignancy (15). Cases tend to occur in older adults (median age ~65–70) and often in males (SEER: 62.5% male) (7). The lag time from first cancer to glioma averages several years, with a median of 4–5 years in the cohorts (6, 7). Given the inability of haematological malignancies to develop typical solid, intracranial metastases, we exclude these cancers from our study.

Data showed that the outcomes for HGG after EXCA were generally poor and mirrored sporadic HGGs. Population data reported a mOS of only about 6 months, respectively 8 months after the diagnosis of EXCA (7, 8). The impact of EXCA on OS also depends on whether it was diagnosed as a first or second tumour. EXCA was considered as a significant risk factor for OS but did not have a statistically significant impact on Brain Cancer-Specific Survival. GBM exhibited the most substantial and statistically significant impact on OS (8). Institutional series tend to show slightly longer survivals. Maluf et al. found a median OS of 14 months (range 1–44) in 21 such patients (5). In one of the largest GBM cohort, patients with prior cancers had a median survival of 13 months versus 15 months without prior cancer, but the difference was not statistically significant (15). Also, our multicentre series reported a median of PFS in GBM of 8.4 months and mOS-GBM of 10.6 months. According to adjuvant oncotherapy, 14 patients were able to undergo Stupp protocol and had mOS of 19.2 months. Eight patients with RT followed by CHT had mOS of 14.6 months. For both the 9 patients who had only RT and the 12 patients without any adjuvant oncotherapy, there was a substantial reduction of mOS to 3.6 and 2.5 months, respectively. One elderly patient with only adjuvant CHT achieved relatively long survival of 14 months.

According to the literature, the standard factors, such as clinic status and age, are the most relevant for eligibility to undergo surgical and oncologic treatment and resulting life expectancy (5). Also, our univariable analyses in this patient group confirmed that traditional prognostic factors (patient’s age, performance status and extent of resection) drive outcome in this specific cohort of GBM patients with previous EXCA. In Maluf’s similar series, the extent of surgical resection was the factor significantly associated with a longer survival (5). In another series, there was no information about the strategy used to obtain histologic diagnosis (including needle biopsy), nor about the surgical radicality (15). When evaluating the postoperative residual GBM volume in our patient cohort, mOS in Class 2A resection was 14.6 months, in Class 2B/3A, it was 8.6 months, in Class 3B, it was 2.7 months. The mOS for patients undergoing aggressive adjuvant oncotherapy in the Stupp protocol was 19.2 months. Based on our data, we may conclude that when patients were able to receive standard therapy for glioma, their survival was comparable to patients without a cancer history. We speculate, that the difference of SEER-based mOS of 6 months after a second primary brain tumour reflects the selection of patients who were eligible for aggressive surgical and oncological treatment (7). We share the same experience, that a history of cancer itself is not an adverse factor if standard treatment is given (5).

Most gliomas that appeared after EXCA were genetically similar to sporadic HGGs. There is no strong evidence of novel genetic alterations shared between the EXCA and the glioma (5). Of course, rare inherited cancer syndromes (e.g. Li-Fraumeni, NF1) can predispose to both gliomas and extracranial tumours, but most reported cases lack such syndromic history. A few gliomas may be related to radiotherapy or chemotherapy, but these are exceptions (16). Generally, the gliomas are considered independent primaries (5). Only Zacharia et al. compared GBMs with and without prior cancers and found some differences in common molecular markers: tumours in the prior-cancer group had more often EGFR amplification (20% vs 12%), MGMT promoter methylation (18% vs 12%) and were typically IDH-wildtype high-grade gliomas (15). In our GBM patients for whom MGMT status was available, approximately three-quarters had an unmethylated MGMT promoter (17/23; survival analysis performed on 22 patients with complete follow-up), which again proved to be a negative prognostic factor. No rare genetic syndrome was detected in any our patient. In our GBM series, a longer OS was proved in the patients with MGMT methylation (mOS = 29.3 months) in comparison to MGMT unmethylated (mOS = 12.0 months).

Reported series (Maluf et al., 2002) showed adverse effect of misdiagnosis on proper treatment strategy (5). Four patients were initially thought to have metastases and received only whole-brain radiation, which delayed the optimal glioma treatment (5). We also recommend managing these gliomas in the same way as EXCA naive GBMs. Again, an adverse overall clinic status caused oncotherapy modification, that yielded limited outcomes. The patients without full therapy had generally poor performance status and/or very advanced prior disease. Although in time of GBM diagnosis, five of our patients had EXCA in a stable state and two patients had EXCA in progression, and during GBM disease two patients had EXCA in a stable state and six patients had EXCA in progression, all our patients succumbed to GBM. All patients with EXCA progression were administered a systematic oncological treatment, however, GBM disease course was faster. And also, there was no evidence that the prior EXCA treatment alone reduces responsiveness to GBM therapy. Although temozolomide is not standard therapy for these extracranial malignancies, preclinical studies have examined sensitisation in pancreatic NET, cervical, melanoma and lung cancer cell lines (18–21).

The potential coexistence of prior EXCA and a new brain lesion poses several challenges. Foremost is a diagnosis. Radiographically, HGG and metastasis often look alike (ring-sign enhance, edema), so reliance on imaging can be misleading. In many of these cases, neuroimaging alone cannot distinguish a metastatic lesion from a primary glioma (22). In the light of new findings, the original idea of ​​EXCA metastasis as a circumscribed lesion in the brain parenchyma, encompassed by pure edema, has been challenged. The existence of a peritumoral infiltration zone has also been demonstrated in brain metastases, which makes it difficult to make a precise diagnosis based on MRI (23). To avoid making a premature judgment based on imaging mimics of EXCA metastases or a primary brain tumour, histological confirmation is needed for accurate diagnosis. Glioma occurring after other EXCA represents a very sporadic event, so careful evaluation (including histopathology) is essential.

Management must also account for the patient’s overall oncologic context. Performance status may be affected by prior therapies, which can influence treatment tolerability. Important is the coordination between neuro-oncologists, neurosurgeons, and the patient’s prior oncology team. If a primary brain tumour is diagnosed, follow-up focuses on glioma progression, however, the history of prior cancer must still inform the overall prognosis and supportive care. Previous radiation fields or chemotherapy regimens may constrain options. Also, exclusion of such patients from trials may limit evidence-based guidance. Clinicians must often extrapolate from general GBM data. The staging and status of the prior cancer can influence goals of care.

## 5 Conclusion

Epidemiologically, only a few of EXCA survivors develop a new HGG. According to clinical features, age of presentation and molecular features, they are typically IDH-wildtype GBMs, mirroring the prognosis of sporadic GBM. Neuroimaging alone cannot distinguish a metastatic lesion from a primary glioma, so surgical biopsy is needed for an accurate diagnosis. Thus, careful evaluation (including histopathology) is essential when a cancer survivor presents with a new brain mass. Standard treatment protocols are recommended for the treatment of GBM patients with EXCA history. Maximal radical and safe surgical resection followed by chemo-radiotherapy in the Stupp protocol is the usual approach for eligible patients. In our patient group, we also confirmed the negative prognostic effect of the postoperative residual GBM volume and the positive prognostic effect of adjuvant oncotherapy. It is crucial that physicians should not dismiss the possibility of a primary glioma in a cancer survivor. Awareness of this phenomenon, as well as multidisciplinary care, is essential for navigating the diagnostic and therapeutic challenges.

### 5.1 Limitations

This study has several limitations. First, selection bias is inherent to a surgical cohort: patients diagnosed by needle biopsy only or managed conservatively were excluded, which favours those with better performance status and surgical eligibility. Second, MGMT promoter methylation status was assessable in only 23 of 48 cases (48%), limiting the power of the strongest molecular finding. Third, the study period (2010–2022) spans three iterations of the WHO classification of CNS tumours (2007, 2016, 2021), and historical diagnoses were retrospectively reclassified. Fourth, no internal non-EXCA control group was constructed from the same registry, so comparisons to sporadic GBM rely on external literature. Fifth, the relatively small subgroup sizes yielded wide 95% confidence intervals, and log-rank p-values should be interpreted with appropriate caution. Finally, the cohort is single-country and may not generalise to populations with different cancer-screening practices.

## Data Availability

The de-identified individual-patient datasets analysed during the current study are not publicly available because they contain potentially identifying clinical information from a small cohort, but anonymised summary data are available from the corresponding author on reasonable request, subject to a data-transfer agreement and approval of the local Ethics Committee.
